# Fabrication of Single-Phase Manganese Oxide Films by Metal-Organic Decomposition

**DOI:** 10.3390/ma14092338

**Published:** 2021-04-30

**Authors:** Kyung-Hwan Kim, Do Kyung Lee, Yun-Hyuk Choi

**Affiliations:** School of Advanced Materials and Chemical Engineering, Daegu Catholic University, Gyeongsan 38430, Gyeongbuk, Korea; 20hwans@naver.com (K.-H.K.); dokyung@cu.ac.kr (D.K.L.)

**Keywords:** Mn_2_O_3_, Mn_3_O_4_, film growth, metal-organic decomposition, solution process

## Abstract

Here, single-phase Mn_2_O_3_ and Mn_3_O_4_ films are successfully fabricated by a facile solution process based on metal-organic decomposition (MOD), for the first time. A formulated manganese 2-ethylhexanoate solution was used as an MOD precursor for the preparation of manganese oxide films. The difference in thermal decomposition behavior of precursor solution in air and inert atmospheres was observed, indicating that the calcination atmosphere is the main factor for controlling the valence of manganese oxide films. Significantly, the solution-coated films on substrates are found to be transformed into single-phase Mn_2_O_3_ and Mn_3_O_4_ films when they are calcinated under air and inert atmosphere, respectively. The film crystallinity was improved with increasing calcination temperature for both Mn_2_O_3_ and Mn_3_O_4_ films. In particular, it is noted that the grains of Mn_2_O_3_ film were somewhat linearly grown in air, while those of Mn_3_O_4_ film exhibited the drastic growth in Ar with an increase of calcination temperature.

## 1. Introduction

Manganese oxides have drawn attention as promising energy storage materials such as rechargeable batteries and electrochemical capacitors as well as environmental catalysts [1,2,3,4,5,6,7,8,9]. The manganese based on a transition metal element with five unpaired electrons can exist in the form of various oxidation states: Mn(IV) oxide (MnO_2_), Mn(III) oxide (Mn_2_O_3_), Mn(III)/Mn(II) mixed-valence oxide (Mn_3_O_4_), and Mn(II) oxide (MnO) [1,8,10]. Such valence flexibility plays a crucial role in determining and optimizing the electrical, electrochemical, and catalytic properties with plenty of opportunities for redox reactions in manganese oxides. However, at the same time, such characteristic creates difficulties in the fabrication of pure single-phase manganese oxides. Thus, a variety of synthetic strategies such as thermal decomposition, exfoliation, permanganate (KMnO_4_) reduction, adsorption-oxidation, and hydro/solvothermal methods have been attempted to obtain pure single-phase manganese oxide nanostructures [10]. Despite a lot of efforts, there are still restrictions on the controllable preparation of pure single-phase manganese oxides, including compositional inhomogeneity, phase instability, and low reproducibility.

The metal-organic decomposition (MOD) technique involves primary synthesis of a stable and homogeneous precursor solution. It provides the benefits of easy composition tuning and high-throughput processing with high metal content, high solubility in organic solvents, thermal decomposition without melting or evaporation, and stability under ambient conditions [11,12]. Moreover, prepared MOD inks can be utilized for printed electronics based on inkjet, microcontact, offset, and gravure techniques, which enable fast, low-cost, and large-scale uniform production of electronic and flexible devices [13,14,15,16]. The difference between MOD and sol-gel methods should be noted. The MOD involves the physical and thermal reaction by the pyrolysis of precursors, while the sol-gel method involves the chemical reaction by hydrolysis and condensation. In the case of complex multicomponent oxides, the control of hydrolysis and condensation is difficult and thus MOD method is preferred. However, to our knowledge, there are no reports of the MOD process for manganese oxides.

In the present work, single-phase manganese oxide films, Mn_2_O_3_ and Mn_3_O_4_, are fabricated by the facile MOD solution route, for the first time. Although manganese oxides are conventionally hard to be obtained in the form of single phase because of their multivalence, the film form gains a competitive advantage in the homogeneous creation of single-phase manganese oxides due to its low activation energy for phase transformation arising from high surface-to-volume ratio as well as its uniform heat transfer. Bulk powders and fine nanoparticles have limits in preparing stable single phases owing to unequal heat transfer and excessive surface reactivity leading to instability in composition and phase, respectively.

## 2. Experimental

### 2.1. MOD Process for the Fabrication of Manganese Oxide Films

**Figure 1:**
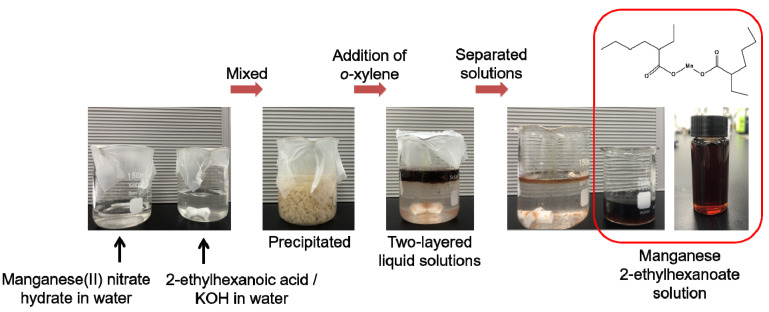
Schematic representation showing the preparation process of manganese 2-ethylhexanoate solution, used as MOD precursor for the fabrication of manganese oxides.

First, manganese 2-ethylhexanoate, Mn[OOCCH(C_2_H_5_)C_4_H_9_]_2_, was synthesized as an MOD precursor for the preparation of manganese oxides. For this, 10 mmol manganese(II) nitrate hydrate (Mn(NO_3_)_2_·xH_2_O, 98% purity, Sigma-Aldrich, Munich, Germany) dissolved in 25 mL deionized water. A mixed solution of 2-ethylhexanoic acid (8 mL) (CH_3_(CH_2_)_3_CH(C_2_H_5_)COOH, 99% purity, Sigma-Aldrich), KOH (3.3 g) (85% purity, Sigma-Aldrich), and 50 mL deionized water was separately prepared. Then, the manganese(II) nitrate hydrate dissolved in deionized water was mixed with the solution of 2-ethylhexanoic acid, KOH, and deionized water under stirring and held for 1 h at room temperature. As a result, solid-state precipitates were formed within the mixed solution. To extract the precipitates, 20 mL *o*-xylene (97.5% purity, Daejung, Siheung, Korea) was added slowly into the solution, leading to the formation of two-layered liquid solutions. The two-layered solutions were separated with the help of a separating funnel and finally, the manganese 2-ethylhexanoate solution was obtained. The series of processes are shown as photographs in Figure 1.

For the fabrication of manganese oxide films, the drop-casting method was used. A cleaned SiO_2_/Si substrate with dimensions of 2 cm × 1 cm, was placed on a hot plate heated at 60 °C. A loading of 100 μL manganese 2-ethylhexanoate solution was dropped onto the substrate using a micropipette. Subsequently, the coated solution was burned out on the hot plate at 200 °C for 2 h in air. Finally, it was calcinated at 400, 800, or 1000 °C for 1 h in open air under ambient conditions or under an inert atmosphere of high-purity Ar (99.999%, flow rate 20 cc/min) with a tube furnace reactor. For the calcination, the samples were heated to each temperature at a ramp rate of 5 °C/min, kept for 1 h, and then cooled naturally to room temperature.

### 2.2. Structural Characterization

The thermal decomposition behavior of solution was analyzed by thermogravimetric analysis (TGA, Q500, TA Instruments, New Castle, DE, USA) from 25 to 1000 °C at a scan rate of 5 °C/min in air or Ar (99.999% purity) atmosphere. The phase and crystallinity of film samples were confirmed by the X-ray diffraction (XRD) method using a D8-Discover (Bruker AXS, Billerica, MA, USA) equipped with a Cu K_α_ source (λ = 1.5406 Å) as well as by confocal Raman microprobe analysis using a Horiba XploRA instrument (Kyoto, Japan). Raman spectra were collected with excitation from the 532 nm line of an air-cooled solid laser. The surface and cross-sectional morphologies of film samples were observed by field-emission scanning electron microscopy (FE-SEM, S-4800, Hitachi, Tokyo, Japan). The cross-sectional micrographs were obtained by observing the cut section of film samples.

## 3. Results and Discussion

To fabricate manganese oxide films through the MOD route, the manganese 2-ethylhexanoate solution was prepared by using the following chemical reactions (1) and (2):

KOH + CH_3_(CH_2_)_3_CH(C_2_H_5_)COOH → CH_3_(CH_2_)_3_CH(C_2_H_5_)COOK + H_2_O (1)

2CH_3_(CH_2_)_3_CH(C_2_H_5_)COOK + Mn(NO_3_)_2_ → 2KNO_3_ + Mn[OOCCH(C_2_H_5_)C_4_H_9_]_2_ (2)

**Figure 2:**
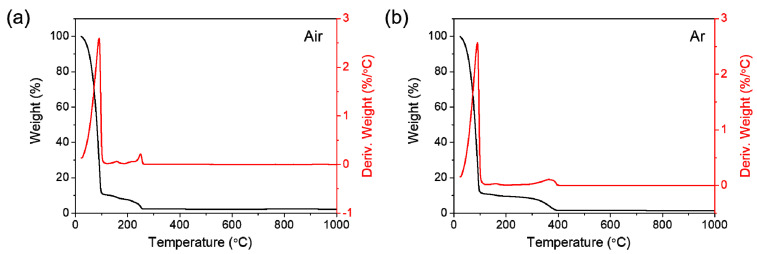
TGA and the derivative TGA curves of manganese 2-ethylhexanoate solution, acquired in (**a**) air and (**b**) Ar.

Thermal decomposition behavior of the prepared manganese 2-ethylhexanoate solution has been investigated by TGA in order to determine MOD processing temperature in ambient air and an inert argon (Ar) atmosphere. The first mass loss, which occurs in a single thermal event until heating temperature reaches around 100 °C, corresponds to dehydration in both air and Ar (Figure 2a,b). The resulting residue is estimated at 11.0 wt%. The difference in the second mass loss between air and Ar is notably detected; the decomposition in air is completed at 255 °C with the final residue of 2.3 wt%, while the one in Ar is achieved at a higher temperature (392 °C) with a smaller amount of final residue (1.6 wt%). The second mass loss is derived from the thermal decomposition of organic compound and solvent in solution. In air, such a mass loss proceeds at a lower temperature due to the facilitation of decomposition by the inflow of atmospheric oxygen. As a result, the formation of manganese oxide residue results from the oxidation reaction by a large amount of flowing oxygen from both solution and air. In contrast, the decomposition in Ar produces a smaller amount of oxide residue, only by oxygen species from solution. Soon after the second loss, the formed intermediate phases were identified by calculating weight losses, which were MnO and more oxygen-deficient MnO_1−δ_ for air- and Ar-calcinations, respectively. Such decomposition behavior hinted that the calcination atmosphere of precursor solution can be the main factor for controlling the valence of manganese oxide products.

**Figure 3:**
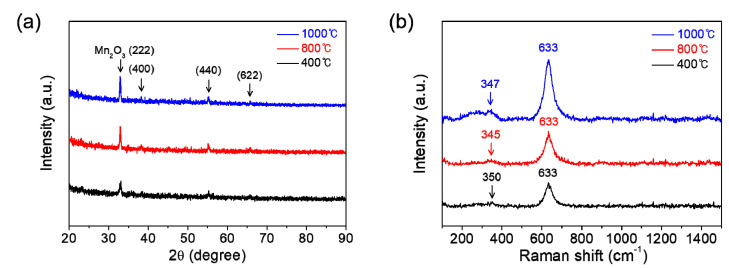
(**a**) XRD patterns and (**b**) Raman spectra of the manganese oxide (Mn_2_O_3_) films prepared at various calcination temperatures of 400, 800, and 1000 °C for 1 h in air.

Figure 3a shows the XRD patterns of manganese oxide films which are coated on SiO_2_/Si substrates and then calcinated at 400, 800, or 1000 °C for 1 h in air. All the films are revealed to be single-phase polycrystalline Mn_2_O_3_, which coincides well with ICDD no. 00-041-1442. The Raman spectroscopy affords useful supplements to XRD for the phase characterization of films. Figure 3b represents the Raman spectra of those films. The main bands at around 350 and 633 cm^−1^, which are commonly observed for each film, can be attributed to asymmetric stretching of bridge oxygen species (Mn-O-Mn) and symmetric stretching of Mn_2_O_3_ groups, respectively [17,18]. The intensities in both XRD and Raman spectra are observed to be gradually increased with an increase of calcination temperature, which indicates the improvement of film crystallinity with calcination temperature.

**Figure 4:**
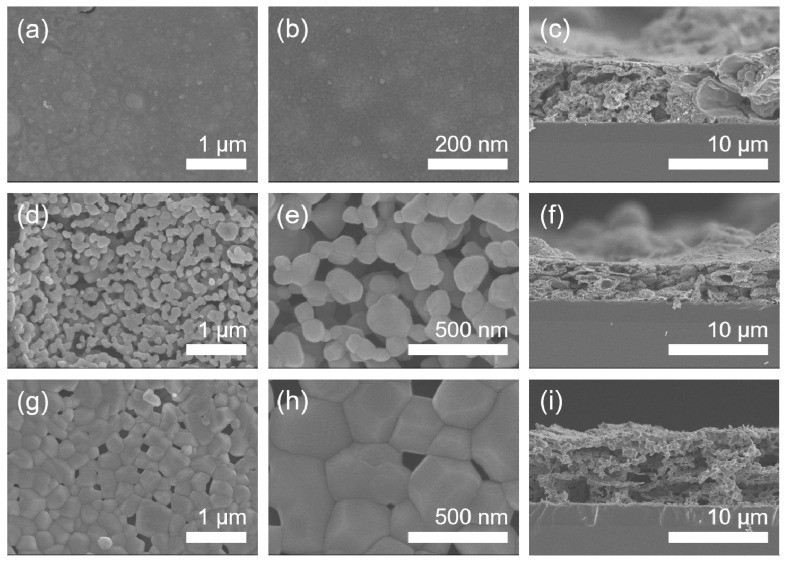
FE-SEM micrographs of the manganese oxide (Mn_2_O_3_) films. (**a**) The low-magnification and (**b**) high-magnification top-view images and (**c**) cross-sectional image of 400 °C-calcinated films on SiO_2_/Si substrates in air. (**d**) The low-magnification and (**e**) high-magnification top-view images and (**f**) cross-sectional image of 800 °C-calcinated films in air. (**g**) The low-magnification and (**h**) high-magnification top-view images and (**i**) cross-sectional image of 1000 °C-calcinated films in air.

The dependence of microstructure on calcination temperature in the Mn_2_O_3_ films calcinated in air has been investigated by FE-SEM as shown in Figure 4. The Mn_2_O_3_ film calcinated at 400 °C in air represents a relatively smooth surface (Figure 4a,b). Under calcination at 800 °C, the film shows a porous particulate morphology with a grain size of about 150 nm in diameter (Figure 4d,e). The calcination at a higher temperature of 1000 °C is found to induce grain coarsening (the grain size of about 330 nm in diameter) and concomitant reduction in porosity (Figure 4g,h). The cross-sectional thickness of the films formed on substrates is observed to be roughly 6 μm (Figure 4c,f,i).

**Figure 5:**
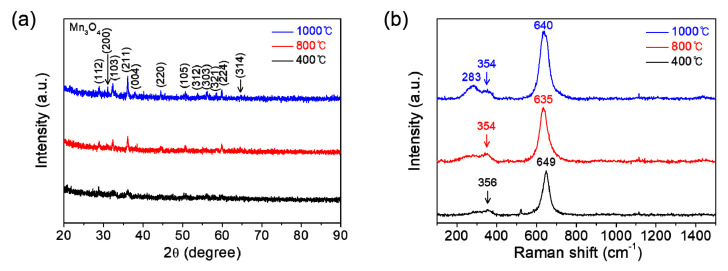
(**a**) XRD patterns and (**b**) Raman spectra of the manganese oxide (Mn_3_O_4_) films prepared at various calcination temperatures of 400, 800, and 1000 °C for 1 h under an inert atmosphere of Ar.

Moreover, we have performed the calcination of solution films coated on substrates at various temperatures under an inert atmosphere of Ar in order to change the stoichiometry in manganese oxide films. Figure 5a displays the XRD patterns of manganese oxide films which are coated on substrates and then calcinated at 400, 800, or 1000 °C for 1 h in Ar. Single-phase polycrystalline Mn_3_O_4_ (ICDD no. 00-001-1127) is clearly confirmed in all the films, which is in stark contrast with that of films calcinated in air (Mn_2_O_3_). Figure 5b exhibits the Raman spectra of those films. The strong peak at around 640 cm^−1^, broad weak bands at 354 and 283 cm^−1^ are consistent with those reported in the literature for Mn_3_O_4_ [19,20,21]. Particularly, the strong peak at around 640 cm^−1^ corresponds to the Mn-O breathing vibration of divalent manganese ions in tetrahedral coordination [21]. Similarly in the case of calcination in air, the peak intensities in both XRD and Raman spectroscopy increase gradually with increasing calcination temperature, also indicating the improvement of film crystallinity with calcination temperature under an inert atmosphere of Ar. Strong XRD characteristic peaks were hard to obtain for the films. It can be speculated that an impurity phase such as Mn_2_O_3_, which goes beyond the analysis resolution of the used XRD and Raman spectroscopy may exist within the films formed by the calcination in Ar. Therefore, more precise high-resolution characterization might be required. Nevertheless, these results can make enough appeal at an industrially available level. Of course, despite the separation, some remaining potassium (K) may exist within the films. However, we could not observe any impurity composition (including K) throughout the energy dispersive spectroscopy (EDS) for the manganese oxide that was burnt out on the hot plate at 200 °C for 2 h in air prior to post-calcination (not shown here). Moreover, since the same precursor was used for the preparation of all films, the difference in the results obtained is not caused by K. While the salts such as K and Na can be used as a flux to promote or hinder the film growth, the amount is too small at an undetectable level for it in this work. Therefore, the effect of K can be ignored.

**Figure 6:**
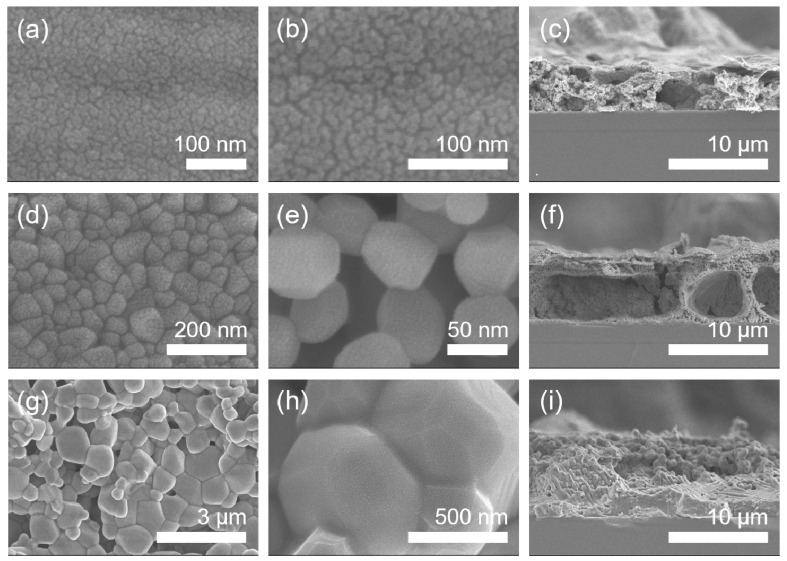
FE-SEM micrographs of the manganese oxide (Mn_3_O_4_) films. (**a**) The low-magnification and (**b**) high-magnification top-view images and (**c**) cross-sectional image of 400 °C-calcinated films on SiO_2_/Si substrates under an inert atmosphere of Ar. (**d**) The low-magnification and (**e**) high-magnification top-view images and (**f**) cross-sectional image of 800 °C-calcinated films under an inert atmosphere of Ar. (**g**) The low-magnification and (**h**) high-magnification top-view images and (**i**) cross-sectional image of 1000 °C-calcinated films under an inert atmosphere of Ar.

**Figure 7:**
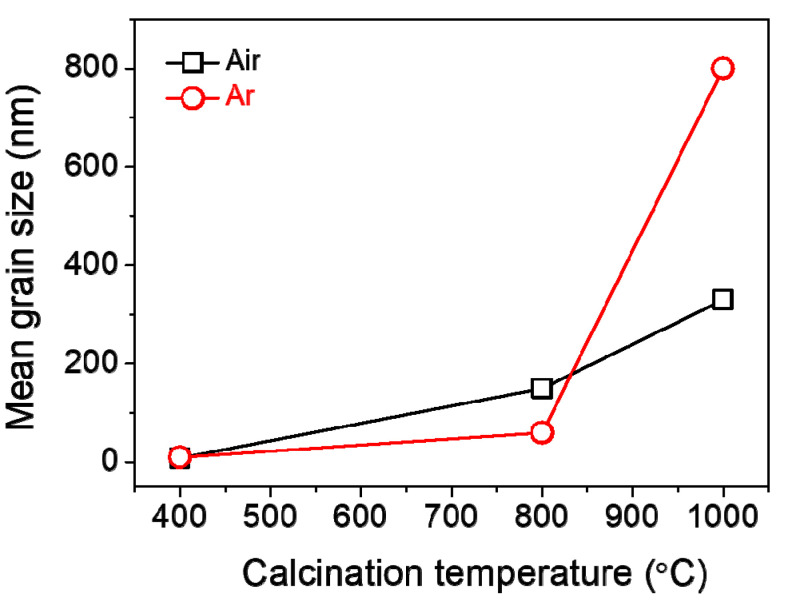
Mean grain size shown as a function of calcination temperature for the films prepared in air and Ar.

The surface and cross-sectional morphologies of the Mn_3_O_4_ films calcinated in Ar are exhibited in Figure 6, which were observed by FE-SEM. As a result, it is found that the grain size increases gradually from about 10 to 60 to 800 nm in diameter as calcination temperature increases from 400 to 800 to 1000 °C in Ar. The cross-sectional thickness of all the films was roughly 7 μm (Figure 6c,f,i). Here, it is worth noticing that the grains of Mn_2_O_3_ film are somewhat linearly grown with increasing temperature under calcination of the MOD precursor solution coat in air (Figure 4), while those of Mn_3_O_4_ film exhibit the drastic growth during calcination up to 1000 °C in Ar, as shown in Figure 7.

In this work, it is well shown that the single-phase films of Mn_2_O_3_ and Mn_3_O_4_ can be prepared via a facile solution process based on the MOD route. In particular, it is noted that Mn(III) oxide and Mn(II)/Mn(III) mixed-valence oxide can be selectively fabricated in air and under an inert atmosphere of Ar, respectively. This technique is expected to be extensively applied to the fabrication of electrodes for various electronic and energy systems.

## 4. Conclusions

The facile solution route based on the MOD process was successfully developed for the valence-controlled fabrication of single-phase manganese oxide films. To begin with, the manganese 2-ethylhexanoate solution was formulated as the MOD precursor for the preparation of manganese oxides. The different thermal decomposition behavior of precursor solution was observed when it was heated under air and an inert atmosphere, i.e., the decomposition was completed with a higher amount of oxide residue at a lower temperature in air, compared with the decomposition under inert atmosphere. Such decomposition behavior hinted that the calcination atmosphere of precursor solution can be the main factor for controlling the valence of manganese oxide products. As a result, the single-phase films of Mn_2_O_3_ and Mn_3_O_4_ could be obtained via thermal calcination of coated films on substrates under air and an inert atmosphere, respectively. The XRD and Raman spectroscopy indicated that the film crystallinity was improved with increasing calcination temperature for both Mn_2_O_3_ and Mn_3_O_4_ films. The FE-SEM micrographs showed that the grains of Mn_2_O_3_ film were somewhat linearly grown in air, while those of Mn_3_O_4_ film exhibited the drastic growth in Ar with an increase of calcination temperature. These results will be utilized for energy and environmental applications.

## Data Availability

Data is contained within the article.
